# The clinical-care focused psychological interview (CLiC): a structured tool for the assessment of cancer patients’ needs

**DOI:** 10.3332/ecancer.2020.1000

**Published:** 2020-01-13

**Authors:** Paola Arnaboldi, Serena Oliveri, Laura Vergani, Giulia Marton, Paolo Guiddi, Derna Busacchio, Florence Didier, Gabriella Pravettoni

**Affiliations:** 1 Psychiatry and Medical Psychology Service (SPPM), Cantonal Socio-Psychiatric Organisation (OSC), Via Tesserete 67, 6942 Savosa, Switzerland; 2 Ticino League against Cancer, Piazza Nosetto, 3, 6500 Bellinzona, Switzerland; 3 Department of Oncology and Hemato-Oncology, University of Milan, Via Festa del Perdono, 7, 20122 Milan, Italy; 4 Applied Research Division for Cognitive Psychological Science, European Institute of Oncology IRCCS (IEO), via Ripamonti 435, 20141 Milan, Italy

**Keywords:** health psychology, patient-centred care, psycho-oncology, psychological intervention, liaison psychology

## Abstract

Integrating the psychosocial perspective in oncology is warranted. Here, we introduce a structured psychological intervention, the clinical-care focused psychological interview (CLiC), to address patients’ needs in the relationship with health professionals, clinical pathway and decision-making process. The perceived utility and feasibility of the CLiC were evaluated in a preliminary sample of 30 patients who were candidates to undergo a radical cystectomy at the European Institute of Oncology, Milan, Italy. Patients reported a very high satisfaction with the interview, because it prepared them for their upcoming surgery by gathering more information about their clinical pathway and allowed them to discover the information they still needed. 30% stated that CLiC helped them to reorganise their thoughts and 36.7% understood the role of psychological intervention in the clinical pathway. Only 20% considered the CLiC useful in helping to build their relationship with the clinical staff. Before an invasive surgery such as radical cystectomy, patients’ need for information regarding the upcoming surgery seems to prevail. Knowing the future consequences and adjusting toward the disease could be paramount for patients in facing uncertainty and they might feel that creating a relationship with physicians could be a secondary issue. However, our data show that a structured psychological intervention such as the CLiC interview can collect important information for patients and healthcare professionals to develop real patient-centred care.

## Introduction

Cancer has a significant impact on patients’ lives, about 25% of newly diagnosed cancer patients present mood disorders, particularly depression and anxiety, which can impair their health-related quality-of-life (HRQoL) and wellbeing [1–5]. The burden of cancer diagnosis and treatment is sometimes worsened by financial needs, the difficulty in finding living solutions to have easier access to the hospital, communication problems with physicians and other practical obstacles that might have a psychological impact [6–8]. Patients experience difficulties in openly facing this type of issue, and can refuse psychological support sessions creating barriers to communication with the medical team and to compliance with cancer treatments [9]. Sexuality issues, in particular, are considered sensitive and taboo in the healthcare context [10], although patients usually suffer negative consequences caused by the disease relating to intimacy with the partner [11–13].

Based on these considerations, we argue that cancer care should involve comprehensive programmes aimed at reducing distress, maintaining a good level of HRQoL, and be able to address most patients’ healthcare-related needs. Many comprehensive care programmes, evaluated in structured trials, are reported to be successful [6, 14]. Nonetheless, recent reviews of the literature about this issue drew the conclusion that evidence-based effectiveness of comprehensive care programmes remains insufficient; they foster more good-quality studies or meta-analysis to show the specific population of patients that would benefit from comprehensive care and evaluate in what moment of their clinical pathway they would need that [15, 16]. We believe that studies could be improved by the application of measures that relate to the patient-defined (personal) perspective of care.

Overall, the implementation of the psychological intervention in the medical routine is still challenging [17]. Different obstacles to psychosocial support have been described in the literature: lack of qualified resources and services; inadequate medical education on the communication between patients and healthcare providers; paucity of empowerment tools available for patients; the absence of psychologists in the ward; the prejudice towards psychological suffering from both patients and healthcare professionals [18, 19]. In such a framework, structured psychological intervention protocols are paramount to integrate psychosocial care in clinical pathways as a routine practice [20–22]. In 2008, the Institute of Medicine (IOM) published its report entitled ‘Cancer Care for the Whole Patient: Meeting Psychosocial Health Needs’ [23], and since then, various initiatives to integrate psychosocial services in cancer care were proposed, such as the screening of patients’ emotional distress and unmet needs [24, 25] and intervention proposals for patients [26]. It is now fundamental to have guidelines and standard protocols to follow [27].

Recently, our research group at the European Institute of Oncology (IEO) and University of Milan proposed to move from a ‘P4 medicine’ approach, which encompasses predictive, personalised, preventive and participatory aspects without considering psychological needs and values, to a ‘P5 medicine’ approach, with the ‘fifth P’ standing for psycho-cognitive aspects related to cancer [28]. This approach aims to include the psychological needs of cancer patients in the organisation and delivery of health services, with a real comprehensive perspective. Following the P5 medicine approach, our proof of concept would describe and report the utility and feasibility of the clinical-care focused psychological interview (CLiC), a psychological tool focusing on patients’ information, communication and relationship needs in the cancer setting. CLiC is based on the following assumptions: psychological distress does not have the same manifestation in all patients, but being a patient, with specific demands, expectations and behaviour as related to the healthcare relationship, is universal and it is indeed a cause of distress. After treating anxiety, depression or sleeping disturbances [29–31], it is fundamental to understand the unique way a patient is experiencing his/her disease. Our proposal is to develop a standardised intervention for information management, decision-making dilemmas and relationship issues shared by cancer patients, independently from the levels of distress related to their psychopathology or the maladjustment experienced.

## New initiatives to integrate psychological support in standard protocols for cancer patients

Italian oncological centres are integrating psychological consultation in their standard protocols. For example, the Humanization of CAncer caRE project (HuCare), an evidence-based medicine (EBM) intervention, aims to improve the psychosocial care of cancer patients in 28 Italian oncological centres [32]. Furthermore, the development and the diffusion of patient-reported outcomes (PROs) [33], a specific measure of patients’ physical status, emotional status and benefits achievable during the different phases of the disease, opened the door to a ‘new era’ of clinical research and care, giving voice to patients’ needs.

From an international perspective, the US National Cancer Institute (NCI) has specifically developed the ‘Patient-Reported Outcomes – Common Terminology Criteria for Adverse Event’ (PRO-CTCAE) an EBM tool to assess adverse treatment effects, as well as perceptions, feelings and mood states from the patient’s perspective in a routine clinical practice [34]. The PRO-CTAE is designed to improve precision and reliability in HRQoL measure of patients during cancer treatment.

As described by a recent systematic review, distressed patients do not often ask for or accept psychological interventions [35]. Nevertheless, psychological involvement in the process of care is an extensive concept that does not stop with the absence of psychological symptoms but aims at the overall well-being of cancer patients. This is what characterises liaison and medical psychology in the broader field of clinical psychology [36]. Therefore, we claim that psychological interventions in oncology and in healthcare settings should not only be delivered after the identification of specific vulnerabilities (full-blown psychological symptoms) but they must address information, communication and relationship needs in the multidisciplinary context.

Here, we introduce a structured psychological intervention to be delivered to all cancer patients and applicable to different phases of their clinical pathway. Independently from the level of distress, psychopathology or maladjustment to illness, the following psychological intervention focuses on patients’ information needs (about the clinical condition and treatments), the decision-making process related to treatments, and the relationship with the health professionals.

## The clinical-care focused psychological interview (CLiC)

We defined the clinical-care focused psychological interview (CLiC) as a specific type of psychological intervention developed to assess information, communication and relationship issues in cancer patients, who may or may not suffer from full-blown psychological problems. Table 1 describes two situations in which IEO inpatients refused the psychological support (despite their high distress), and how the CliC helped to figure out their needs during their hospitalisation.

This intervention can be applied to different multidisciplinary care settings such as: a) pre-hospitalisation for a highly demanding procedure, e.g., major surgery; b) enrolment in clinical trials; c) decision-making about prophylactic intervention and d) the transition to the end of life care.

The interview has a standard structure so that it can be replicated in a different healthcare setting (see Table 2), and it usually takes 30–45 minutes.

As described in Table 2, the CLiC starts with an introductive and descriptive section that has two purposes: a) to reassure patients who might have refused to meet a psychologist, e.g., because they are afraid to be considered psychologically impaired or because they are embarrassed to talk about emotions and intimate issues; and b) to introduce psychosocial care as a wider concept that goes beyond the medical setting, addressing the subjective illness experience and starting the process of patient empowerment. The purpose is to involve patients in collaborating with the medical team and participating in the decision-making process about the therapies.

The second part of CLiC addresses the relationship issues, trying to stimulate reflections about patient–physician communication and interaction.

Bowlby’s description of attachment considers illness as often accompanied by an increased need for caring others [37] that, as described in the literature, may interfere with the patient’s relationship with the healthcare providers [38, 39].

The relationship between healthcare provider and patients is an essential component of the treatment and is based on good communication [40]. Good communication and, consequently, a good relationship can have different positive outcomes: patient’s satisfaction with care [40], better adherence to the treatment [41], support in emotion regulation [42]. Patient–physician communication can be a useful instrument in identifying patient’s needs and it can bring to a better comprehension of medical information [43].

The third part of CLiC addresses information handling and patient’s decision-making processes. Its theoretical background lays in the empowerment model, as described by Tengland [44]. Empowerment is a multifactorial construct, and different factors have been enlightened during the years [45]. The concept of empowerment refers to the process of involving the patients in their care decisions and give them a higher perceived control over the situations. In healthcare settings, empowerment is possible through the promotion of active information management.

Nevertheless, in current clinical practice patients are rarely asked if they are satisfied with the information provided and about their preferred role in the decision-making process and interaction with their physicians. At the end of the interview, patients receive a reformulation and they have the possibility to discuss the contents of their conversation with the psychologist. They may also decide if they want their needs to be reported to the healthcare equipé to improve the quality of care.

In this contribution, oncological patients received the CLiC when they were candidates for a major surgical procedure, at the moment of pre-hospitalisation. Soon after, they were invited to complete a short interview, to assess patients’ satisfaction and perceived utility of the intervention. We obtained the approval for this pilot investigation from the Institutional Review Board of the IEO.

We want to clarify that CLiC mainly aims to determine cancer patient’s needs regarding information, communication and their relationship with the medical equipé, not to deeply elaborate their psychological suffering. Psychological suffering deserves to be treated with personalised sessions of professional support during hospitalisation.

## Methods

### Preliminary evaluation of patients’ satisfaction and perceived utility of CLiC

Perceived utility and feasibility of the CLiC were evaluated in a preliminary sample of 30 patients candidate to undergo a radical cystectomy at IEO, who showed high distress on the distress thermometer [46] (a one-item, 11-point Likert scale that ranges from 0, no distress, to 10, extreme distress, and the cut-off considered was >5 points for reporting a high distress), submitted during pre-hospital admission. During pre-hospitalisation, patients are informed by the case manager nurse that they will receive a clinical-care focused psychological interview together with their other appointments (Electrocardiography ECG, pre-anaesthetic interview, surgical visit and blood test). Besides, our sample of patients with significant level of psychological distress was asked if they would have accepted, in addition, a psychological support for their distress, but they refused at the moment of enrolment for this study. The informed consent was provided and obtained by all the participants of this survey. Patients mean age was 67 (41 minimum age–86 maximum age) and they were predominantly males (85% males versus 15% females). They were invited to complete a six-item questionnaire assessing:

- The level of perceived satisfaction (Visual Analogue Scale (VAS) from 0, not satisfied, to 100, completely satisfied).
- Perceived utility of CLiC (multiple-choice question).
- Perceived efficacy of CLiC for preparing the patient to surgery (VAS scale from 0, no efficacy, to 100, extreme efficacy).
- Perceived efficacy of CLiC for the relationship with the physician (VAS scale from 0, no efficacy, to 100, extreme efficacy).
- Importance in sharing personal contents, such as sexuality, with the medical team (VAS scale from 0, not important, to 100, extremely important)
- Perceived efficacy of CLiC for sharing personal contents, such as sexuality, with the medical equipe (VAS scale from 0, no efficacy, to 100, extreme efficacy).
- Results of VAS scales are reported in Table 2.

Participants showed very high satisfaction with the interview (mean score of 86.11 on the VAS scale), perceiving CLiC was very useful for preparing them to overcome surgery (mean score of 82.5) and in building the relationship with the physician (mean score of 85.0). They considered CLiC moderately important for sharing personal contents such as sexuality. Means scores were 72.67 in the question regarding the importance to share issues about sexuality with the medical equipe, and 79.47 regarding the usefulness of the CLiC in addressing these specific and personal needs.

**Figure 1.**
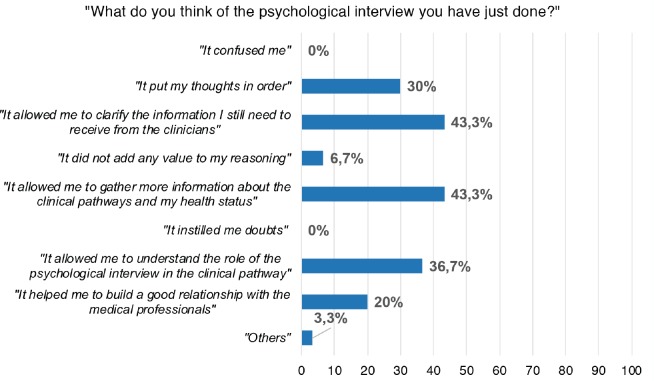
Aspects selected by patients concerning the perceived utility of CLiC.

Another item specifically asked the patients about their perceived utility of CLiC, offering them a multiple answer choice. Answer frequencies are represented in Figure 1.

None of the patients stated that the CLiC caused confusion or instilled doubts, and only 6.7% of the participants answered that the interview did not add anything to their reasoning. 43.3% of the patients answered that CLiC helped them in gathering more information about the clinical pathway and surgery, and to understand the information they still lack from clinicians. 30% stated that CLiC helped them to reorganise thoughts and 36.7% to understand the role of the psychological intervention in the clinical pathway. Unexpectedly, only 20% considered the CLiC useful in helping to build the relationship with the clinical staff. Finally, only 3.3% of participants specified that CLiC could be useful for other aspects of the care.

To complete the overall picture of the patient’s satisfaction for the CLiC interview and its utility, we report that after the interview about 30% of patients requested further psychological support (9/30 patients).

## Discussion

With this contribution, we introduce the clinical-care focused psychological interview (CLiC), providing preliminary evidence of its acceptability and feasibility in a sample of 30 patient-candidates to radical cystectomy. One of the main CLiC aims is to overcome the barriers in patients’ adherence to psychological intervention in oncology. Furthermore, our results could provide a deep understanding of this instrument and its applicability in cancer care. Patients declared a high overall perceived satisfaction with the interview, reporting CLiC as a very useful tool for their specific situation; since they were all pre-hospitalised patients, candidates for radical cystectomy, they perceived CLiC as very helpful in dealing with the upcoming surgical procedure. Moreover, none of the patients expressed negative comments on CLiC (confusion or doubts caused by the interview). These results must be considered jointly with other important data: despite reporting a high level of distress, before being administered with CLiC, the majority of our sample stated that they wouldn’t accept any psychological consultation for their distress. Nevertheless, after CLiC administration, patients recognised the utility to have a discussion with a professional psychologist and were satisfied with this level of care.

Recalling the CLiC structure, the first section introduces the psychological intervention to the patient as an essential part of routine multidisciplinary care, and it tries to overcome prejudices and shame related to psychological suffering, which are still too common [47]. When asked about CLiC utility, nearly 40% of our sample endorsed these aspects, stating that CLiC allowed them to understand more about the role and utility of the psychological assessment in the whole clinical pathway.

In the second section, CLiC focuses on the assessment of patients’ relationship with the physicians and their communication needs. Effective patient–doctor communication has a lot of benefits [43] and different scopes: encourage information exchange, facilitate decision-making process, and the establishment of a good relationship in the doctor–patient dyad [48]. Our sample perceived CLiC as very useful in helping them build a relationship with the physician (mean score VAS scale 85/100). Nevertheless, in the second multiple choice question only 20% of patients endorsed the option that the CLiC helped them in ‘building a good relationship with the medical professionals’. This data could be interpreted, considering the peculiarity of our sample composed by pre-hospitalised patients who were candidates for radical cystectomy. Indeed, before such invasive surgery, patients’ need of information regarding the upcoming surgery, the future consequences and adjustment to the disease could be paramount and patients may feel that creating a relationship with the physicians could be a secondary issue, compared to the amount of information they want to gather for facing uncertainty related to their clinical pathway. Furthermore, our sample was composed mostly by male patients with a mean age of 67. The literature shows that male patients preferably concentrate on informational issues rather than on relational ones [49], and that ‘older’ patients tend to be offered less participatory involvement in their care than patients younger than 30 [50]. In this context, CLiC may represent a ‘safe space’, without time constraint, in which patients may feel validated and respected regarding their needs and preferences (e.g., ‘some patients prefer to be well informed, others prefer that a caregiver is informed in their place…’, ‘it is very important for the equipe to understand how you are feeling’).

To confirm what was observed above, the most valuable characteristic of CLiC, as judged by the patients, was related to its utility in gathering more information regarding the clinical pathway and, in this case, the upcoming surgery (mean score on the VAS Scale 82.5 referred to the question `How much the psychological interview helped you to deal with the surgical procedure?’, see Table 3, and 43.3% of patients choosing `helped me in gathering more information about the clinical pathway and the surgery’ in the multiple-choice question, see Figure 1). Finally, CLiC also evaluates patients’ needs and intentions to share personal issues and experiences with the medical equipe. Usually, one main personal issue dealing with patients candidate to radical cystectomy is intimacy and sexuality [51]: both men and women that undergo radical cystectomy are affected by changes in sexual functioning, which can make intimate relationships very difficult [52]. Moreover, sexuality is often considered as an unresolved issue in cancer care [53]. Thus, we asked our sample if they consider sexuality as an important issue to address with the medical equipe, and the utility of CLiC in addressing it. With moderately high mean scores, our sample considers sexuality concerns to be an important issue, that needs to be shared with the health professionals, and CLiC is considered to be a valid tool to facilitate this intimate conversation (mean score on VAS Scale 79.47).

As discussed before, CLiC is meant to be administered to all cancer patients, in different phases of the clinical pathway, who may or may not suffer from psychological problems, in various multidisciplinary care settings—this instrument has a very wide applicability. The sample of patients enrolled for our preliminary evaluation, composed of patients who were candidates for radical cystectomy (prevalently males), with a high level of distress during pre-hospitalisation, was very specific. The aim of our contribution is to foster dedicated trials in the wider context of cancer care and with different types of cancer patients, to have scientific evidence of the efficacy and utility of CLiC.

## Conclusion

Our proof of concept shows the potentiality of CLiC as a new structured psychological intervention to develop patient-centred approaches in medicine.

The most valuable aspect was CLiC’s utility in dealing with the patients’ overcoming clinical steps, in building a relationship with medical professionals, in gathering more information regarding the personal clinical situation and in understanding the role of the psychological interview in medical care.

Comprehensive care programmes have to be included in cancer care, and psychosocial interventions need to be integrated into clinical routine practice, with the purpose of assessing patient’s needs and addressing overall wellbeing.

CLiC is shown to be a useful tool in addressing patient’s needs and in overcoming some barriers to the psychological interventions in cancer care.

## Conflict of interest

The authors declared no conflicts of interest with respect to the authorship or the publication of this article.

## Funding

The authors declare they had no funding source for this manuscript.

## Figures and Tables

**Table 1.** IEO inpatients refusal of psychological support and CLiC assessment.

Clinical case examples	Psychological support proposal	CLiC
M. is a 62-year-old woman who was diagnosed with bladder cancer. She is a professor, describing herself as self-confident and determined. In relationship with physicians and nurses, they perceive her as detached, devaluating, not interested. As a counterpart, they feel not useful and angry.	She scored 6 at the distress thermometer based on anxious symptoms. When proposed, she refused a psychological consultation to treat and better assess her symptoms.	CliC helped in clarifying that her behaviors were linked to a relational pattern in which she toils to ask for help. Referring to this aspect to the equipe, helped in creating a collaborative environment and helped professionals to have a more proper way to interact with such a patient.
S. is a 49-year-old hairdresser with bladder cancer. During her twenties, she was diagnosed with a borderline personality disorder. She takes antidepressants and mood stabilisers. She is extremely anxious and demanding and makes |professionals feeling overwhelmed and powerless as if all they care was never enough.	She refused a psychological consultation and support claiming having had a long psychiatric history.	She was introduced to the CLiC in which she opened about her need for information and her feeling of helplessness and not cared for by others. Sharing these contents with equipe members helped them in mobilising resources to better assist the patient lowering anxiety and powerlessness.

**Table 2.** Structure of the clinical care focused psychological interview: excerpts and purposes.

Excerpts	Purposes
Have you been informed by my colleagues that we would meet today?	Integrating CLiC into routine multidisciplinary care (the mental healthcare professional as part of team intervention). Going beyond the concept of psychology professionals as mere consultants, outside the medical équipe.
The reason why we are meeting today has nothing to do with an alleged psychological disorder…	Overcoming prejudices relating to psychological suffering. Reassuring patients about their psychological integrity.
We are meeting today to discuss together some important issues about your clinical pathway, and to assure you a good interaction, communication and information exchange with medical professionals.	Creating a culture in healthcare, concerning the importance of information exchange, communication, and the patient–physician relationship.
You are dealing with a complex process of care, which involves several medical professionals. Your specific needs, regarding communication, information exchanges, interaction during the clinical pathway are paramount for the medical équipe. Would you like to share your personal experience when dealing with an issue, in terms of how you behave when you are in need of help? Do you feel some discomfort?	Allowing patients to recognise the importance of their needs during the clinical pathway, first underlying the important role that communication and interaction have for the équipe members
Are you satisfied with the information you gathered about your present clinical situation? Some patients prefer to be well informed, others prefer that a caregiver is informed in their place. This preference can vary through the different phases of the clinical pathway. It is very important for the équipe to understand how you are feeling. Would you like to talk about it?	Creating a culture around the role of being well informed and participate in the decision-making process, respecting patients’ intraindividual and interindividual preferences.

**Table 3.** Patients’ evaluation of satisfaction and perceived utility of CLiC.

Item	N	Mean	St. deviation
*‘How much is your perceived satisfication regarding the psychological interview you have just done?’*	27	86.11	17.06
*‘How much the psychological interview helped you to deal with the surgical procedure?’*	30	82.5	18.65
*‘How much the psychological interview helped you to build a relationship with your physician?’*	30	85	18.19
*‘The surgical procedure you are undergoing have some effects that may negatively impact on your quality of life , in which sexuality is included. How important is, in your opinion, the possibility to share this issue with the medical equipe?’*	30	72.67	30.67
*‘Do you think that the psychological interview could facilitate communication process between patients and medical equipe regarding sexuality issues?’*	30	79.47	29.32
